# Extended Reality—New Opportunity for People With Disability? Practical and Ethical Considerations

**DOI:** 10.2196/41670

**Published:** 2024-02-13

**Authors:** Karen Stendal, Rosemarie D L C Bernabe

**Affiliations:** 1 Department of Business, Marketing and Law University of South-Eastern Norway Honefoss Norway; 2 Centre for Medical Ethics University of Oslo Oslo Norway; 3 Department of Optometry, Radiography and Lighting Design University of South-Eastern Norway Kongsberg Norway

**Keywords:** extended reality, virtual worlds, virtual reality, disability, practical, ethical, technology, virtual, reality, development, research, challenges

## Abstract

Since the introduction of virtual environments in the 70s, technologies have moved through virtual reality, mixed reality, and augmented reality into extended reality (XR). This development is promising for various groups. Previous research has shown people with disability benefiting from using technology in social and professional settings. Technology has offered people with disability the opportunity to communicate, interact, participate, and build new relationships. However, we do not know what impact XR has or will have and whether it will offer new opportunities for people with disability. This paper aims to indicate potential opportunities and challenges afforded by XR to people with disability. We offer reflections on the opportunities as well as the ethical considerations needed when introducing immersive technologies to a marginalized group.

## Introduction

We live in a society where technology in many ways covers our needs, which may also help people with disability in various ways [1]. Extended reality (XR) is moving into various areas of our lives and in areas of research such as rehabilitation [2], education [3], and tourism [4]. What impact will this technology have on people with disability? We look into XR head-mounted displays, which can become mainstream.

## What is XR?

Various technologies used for social interaction have been in rapid development. Virtual worlds have, in countless versions, existed since the late 1970s [5-7]. Today, we see XR as the latest example of this technology.

XR is an umbrella term used to describe technologies such as virtual reality, augmented reality, and mixed reality [8].

XR has been described as technology that blurs the boundary between the physical and virtual worlds and enables users to experience a sense of immersion [8]. The term immersive refers to “the degree to which a virtual environment submerges the perceptual system of the user in computer-generated stimuli” [9]. Being immersive means that individuals wearing head-mounted technology will use several senses, such as vision and hearing. This way, the physical world will be “shut out” and the individual will be totally immersed in XR. This gives a sense of presence, of “being there,” in a way not experienced through virtual technology on a screen [10].

Research exploring XR for people with disability is of current interest since previous research found virtual worlds to be promising for people with disability [5,11-13]. Previous research has also included virtual reality for people with disability; however, it has in many cases involved virtual reality on a screen from a distance [14,15], where the user sees themselves in the virtual environment. The question continues to be whether XR offers the same or new opportunities for people with disability, as called for by previous literature reviews [16,17].

## New Opportunities Come With Practical and Ethical Challenges

People with disability experience various challenges, such as low self-esteem, a lack of support, stigma or discrimination, and a low degree of employment [18], not to mention the persistent exclusion or distortion of the disability narrative and experience in technological development [19]. Being treated as equals and having the same access to social and professional arenas as nondisabled peers are important to minimize or eliminate these challenges. Previous research has introduced virtual worlds as the technology to ensure this [20], and XR is the natural next step.

XR promises to empower people with disability by changing the way we learn, accomplish tasks, and interact with each other [16,21]. Being able to partake in activities not possible in the physical world affords a new opportunity for participation [21] through XR. The technology offers the possibility to visit physical world locations through a headset and ensures a fully immersive experience of the visiting location. Adding haptic technology—a feedback technology—enables a sense of touch by applying forces, vibrations, and motions upon the user [22], which further enhances the immersive experience.

The promises of this technology are abundant. However, there are challenges for people with disability, such as the effect of sensory stimuli, the comfort of wearing the technology, the visual and other physiological side effects of the technology [23], or the emotional outcomes of using XR. Part of being immersed in the technology involves a high level of sensory stimuli [8]. For instance, using a head-mounted display may lead to feeling motion sickness [8]. These results are based on users without disabilities, however, and we have little knowledge about how this technology affects people with disability when they want to use the technology [16]. In addition, there is little knowledge about how vision impairment affects and may further be affected by the use of XR [23]. This technology is made for young adults with perfect vision, which may be a challenge for many people with disability [23]. Thus, there is a need to explore who can take advantage of the technology based on a vision perspective.

Further, previous research has explored the emotional outcomes using XR [8]; however, this is an underexplored area for individuals with disabilities. There are positive emotional outcomes, such as pleasure, fun, happiness, confidence, and hope. In addition, negative emotional outcomes were identified, such as boredom, anxiety, depression, tension, fear, anger, and rage [8]. How these emotional outcomes will affect people with, for example, an intellectual disability should be explored further. XR, similar to other technologies that display avatars, does not sufficiently display body language, such as facial expressions, which makes situations more difficult to understand. Previous research has also stated that people with Asperger spectrum disorder are unable to transfer skills learned in the virtual world to the physical world [24]. Clearly, more research is also needed in this area.

As with any powerful technology, XR has its risks, several of which are relevant for users with disability. For example, XR technology can be highly immersive such that (prolonged) use can lead to social dissociation [25]. This means a user can be so emotionally involved in XR leading to detachment from one’s physical reality–based social relationships. There is also the related risk of addiction [26]. The propensity of individuals with disability to this risk is yet to be explored. At the same time, we have very good reasons to believe that individuals with, for example, specific types of psychological disabilities may be more vulnerable to this risk than others. On top of the question of how they are vulnerable, there is the need to explore what technological safeguards must be put in place to lessen this risk.

Still within the topic of risks is the growing concern for deep behavioral manipulation, deep fakes, and malicious designs in XR. XR’s power lies in its capacity to separate one’s sensory perceptions from the physical world, effectively creating virtual, mixed, or augmented reality [27,28]. When this power is coupled with malicious intent, then the use of XR runs the risk of deep behavioral manipulation, deep fakes, and malicious designs. Especially in the case of individuals with psychological disabilities, but also for other users who may be considered relatively more vulnerable to such malice, the bigger question of what regulatory and technical safeguards must be in place is yet to be answered [25,26,29]. In addition, current metaverses for XR do not consider inclusion and accessibility for people with disability, which also should be considered for future research [30].

By design, XR favors the young White male population without physical or psychological disabilities [31]. This raises the question of how XR can be adapted for universal use, that is, to include as many individuals with disabilities as possible as potential users. This could mean several things such as redesigning hardware and software using the principles of universal design and human-centered design [32] while also considering the special needs of this population for the protection of privacy autonomy, and against the misuse of personal data.

XR technologies bring with them a lot of promises and potential opportunities for individuals with disability. But, as with other potent technologies, some challenges and risks require more research and regulatory attention. There is a need to have a closer look at these emerging technologies, ideally with and by people with disability [19]. There is a tradition of exploring new technology through able-bodied users and making assumptions about the abilities needed to use the technology. There is a need to know the affordances offered by XR to people with disability. Affordances help us understand the technological and human abilities needed to use the technology [21,33] and give us a direction to ensure that more people can take advantage of the technology. In addition, more research is needed on how XR can be used as a support tool in the treatment of people with intellectual and multiple disabilities [34].

## Conclusion

For those who are able, XR may offer many new opportunities. There is, however, a need to understand the limitations, risks, and challenges of the technology. Compared to virtual worlds on a screen, XR may affect multiple senses for the individual, and this may have implications for people with disability. As discussed earlier, they may experience overstimulation, sound issues, or motion sickness or be subjected to risks such as addiction, unintended visual and other physiological effects, malicious design, and privacy issues. Although positive or negative experiences are not exclusively related to people with disability, the potential benefit from XR for people with disability could be significant. By conducting research involving people with disability, we ensure a nuanced picture of the abilities needed to use the technology, without making assumptions.

Future research should explore how people with disability experience XR for several reasons. This technology may empower and enable people with disability to connect increasingly to society and have access to learning environments and social arenas. Through exploring the affordances offered by XR, we may gain knowledge about what facilitating conditions will allow people with disability to take advantage of innovative and new technology.

## Abbreviations

XR: extended reality
